# Ameliorating effect of erythropoietin in a severe case of COVID-19: case report

**DOI:** 10.11604/pamj.2022.43.129.35014

**Published:** 2022-11-09

**Authors:** Mais Mamoon Maufak, Gulfaraz Khan, Ani Purushothaman, Fiaz Ahamed, Mahir Khalil Jallo, Muhammad Rizwan, Sangeeth Kumar, Ahmed Almansouri, Taleb Mohamed Almansoori, Megha Thottappillil Ouseppachen

**Affiliations:** 1 Intensive Care Unit, Thumbay Hospital, Ajman, United Arab Emirates,; 2 Department of Medical Microbiology and Immunology, College of Medicine and Health Sciences, United Arab Emirates University, Al Ain, United Arab Emirates,; 3 Department of Internal Medicine, Gulf Medical University and Thumbay University Hospital, Ajman, United Arab Emirates,; 4 Department of Infection control, Thumbay University Hospital, Ajman, United Arab Emirates,; 5 Department of Pulmonology, Thumbay University Hospital, Ajman, United Arab Emirates,; 6 Department of Radiology, College of Medicine and Health Sciences, United Arab Emirates University, Al Ain, United Arab Emirates

**Keywords:** COVID-19, Severe acute respiratory syndrome coronavirus-2, erythropoietin, acute respiratory distress syndrome, case report

## Abstract

The COVID-19 pandemic is arguably one of the greatest public health crises since the 1918 influenza pandemic. Although several vaccines have been approved and rolled out, effective antiviral treatment options are very limited. Here, we present a case of severe COVID-19 that failed to respond to the standard interventions and continued to deteriorate. On day 22 of his illness, after informed consent, the patient was administered 4000IU of erythropoietin (EPO) subcutaneously, in the hope of improving his O_2_ saturation. Positive response was observed in the patient within 24 hours. This prompted us to continued EPO treatment for a total of 42 days until full recovery and discharge. Our findings warrant further studies to ascertain the use of EPO in severe cases COVID-19.

## Introduction

Severe acute respiratory syndrome coronavirus-2 (SARS-CoV-2), the cause of COVID-19 pandemic, continues to have major impact on the health of individuals worldwide. In spite of widespread COVID-19 vaccination campaigns, the number of new cases and associated deaths are still a major concern, particularly due to new variants and waning immunity. Males, those above 60 years of age, and individuals with comorbidities are particularly at high risk of developing severe disease and dying from the infection [1]. Unfortunately, there are very few approved antivirals for the treatment of COVID-19. Here, we report positive outcomes following the use of recombinant human erythropoietin (EPO) as an adjuvant therapy in a case of severe COVID-19 infection.

## Patient and observation

**Patient information:** a 43-year-old man, with no known co-morbidities, developed flu-like symptoms on 8^th^ Feb 2021. He tested positive for COVID-19 on 11^th^ Feb, and was quarantined at home. His symptoms progressed and he was admitted to Thumbay Hospital Academic Health Center of Gulf Medical University on 18^th^ Feb with fever (37.4°C), cough, breathlessness and diarrhea.

**Clinical findings:** on examination, he was found to be confused, disoriented, tachypneic (34 breaths per min), and hypoxic (O_2_ saturation 74%). His heart rate was 74/min and blood pressure 130/80 mmHg. His chest findings on auscultation indicated bilateral mid and lower zone coarse crepitations.

**Figure 1:**
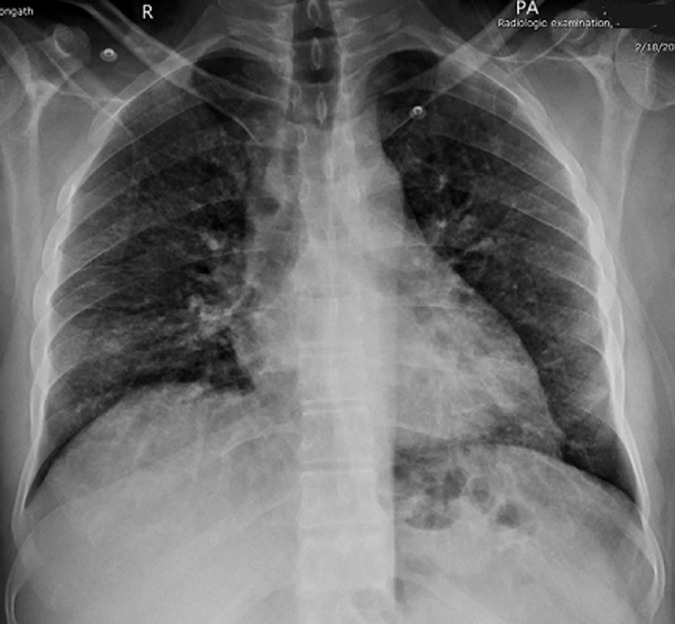
initial chest X-ray taken on 18.2.21 showed bilateral ground glass opacities (GGO) predominantly in the mid and lower zones; no mediastinal abnormality is noted and both costophrenic angles are clear

**Figure 2:**
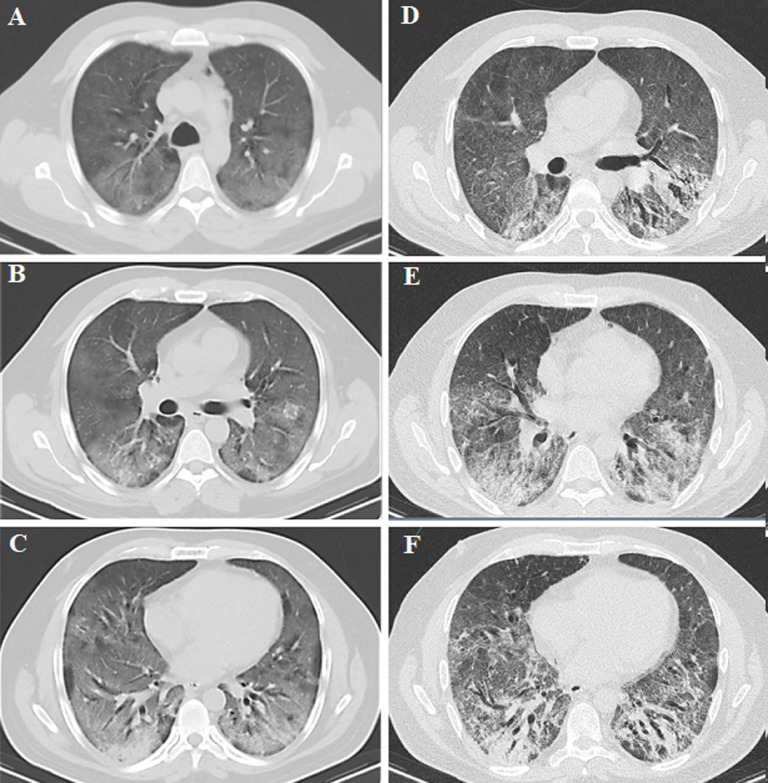
(A, B, C) chest CT demonstrating bilateral ground glass opacities (GGO) involving all lobes of both lungs with consolidations and mild bronchiectasis mainly in the superior segments of the lower lobes of both lungs; halo sign involving the anterior segment of the left upper lobe with bilateral scattered areas of air rapping and septal thickening consistent with COVID-19; (D, E, F) chest CT indicating architectural distortion with traction bronchiectasis and focal areas of consolidations in the superior segments of the lower lobes of both lungs; peripheral GGO in all lobes of both lungs with smooth interstitial thickening suggesting progressive fibrosis following COVID-19

**Diagnostic assessment:** laboratory investigations revealed Hb 13 g/dl, WBC count 4,500/µL, platelet count 234,000/µL, C-reactive protein (CRP) 187 mg/L, S. Creatinine 1.45 mg/dl, ALT 116 U/L, D-dimer 575 ng/ml and S. Ferritin level 1138 ng/ml. Hematocrit level was 38% and transferrin saturation was 43%. Chest X-ray (Figure 1) taken on 18^th^ Feb, and chest CT (Figure 2 (A, B, C)) taken on 27^th^ Feb, revealed bilateral ground glass opacities (GGO).

**Timeline and therapeutic interventions:** the patient was moved to intensive care unit (ICU) and was kept on non-invasive ventilation (NIV) (continuous positive airway pressure (CPAP) mode) with FiO_2_titrated to maintain oxygen saturation of 94%, as he did not tolerate high flow nasal cannula. His medications, as per our hospital COVID-19 protocol algorithm, included antiviral (oral Favipravir), steroids (dexamethasone, later changed to Solumedrol), anticoagulation (Clexane), antibiotics and supportive medication, including vitamins. In view of his persistent severe hypoxia (P/F ratio less than 100) and worsening inflammatory markers (CRP, ferritin, LDH), he was given two doses of Tocilizumab (a monoclonal antibody against IL-6) on day 0 and day 16 of his admission.

Despite the above treatment, the patient continued to have hypoxia on maximal FiO_2_(100%), with arterial blood gas showing P/F ratio of less than 100. At this point (day 17^th^ of admission), his medications included: Meropenem, Levofloxacin, Doxycycline, Tadalafil (for pulmonary hypertension), Diltiazem, Diuretics (furosemide, later amiloride and hydrochlorothiazide), Vitamin C, zinc, Vitamin D, Pantoprazole, Ursodeoxycholic acid (for raised transaminitis secondary to COVID-19 infection and/or antiviral medications, as per our local protocol), insulin movorapid, antitussive syrup, nebulizations albuterol, ipratropium bromide and budesonide, acetylcysteine sachet, Ivabradine (for inappropriate sinus tachycardia), decongestant nasal spray, intravenous multivitamins and intravenous iron (total 5 doses).

Follow-up chest CT on day 17^th^ of admission revealed architectural distortion with traction bronchiectasis and focal areas of consolidations in the superior segments of the lower lobes of both lungs along with bilateral peripheral ground glass opacities (GGO) (Figure 2(D, E F)). The patient was at high risk of clinical deterioration, requiring intubation and mechanical ventilation. Echocardiography revealed pulmonary hypertension, with end systolic pulmonary artery pressure (ESPAP) of 65mmHg. At this stage, we felt the need to go beyond the usual treatment in order to delay intubation and to bridge the critical time period of hypoxia, until the patient´s body mechanisms could compensate. We reviewed the literature and found evidence of prophylactic use of erythropoietin (EPO) to improve oxygenation [2-4]. Following evaluation and discussion, it was decided to enhance the patient´s blood oxygen carrying capacity, but maintain his hematocrit to below 45%. The patient´s starting hematocrit level was 38% and transferrin saturation 43%. Hence, we had adequate window to administer EPO. On day 22 of his admission, after obtaining informed consent, 4000 IU EPO was administered subcutaneously. Based on published literature, we expected improvements within 2-4 weeks. However, this patient showed improvement on the second day. After this positive initial response and encouraging feedback from the patient, we continued administering EPO every few day (day 22, 29, 32, 36, 40, 43, 46, 50, 57 and 64 of admission). Thus, a total of 10 doses of 4000 IU were given over a period of 42 days. Patient´s serum EPO level was checked to ensure it was within normal limits.

**Follow-up and outcome:** in the days following EPO treatment, the patient´s clinical condition gradually improved, as indicated by the increase in arterial PO_2_, reduction in oxygen demand and respiratory rate, and patient´s ability to talk and eat. The patient also felt better in terms of energy and physical activity performance. He continued to participate in physiotherapy sessions. Objectively, follow-up echocardiography also showed improved pulmonary arterial pressure (PAP). His COVID-19 PCR repeated on 25^th^ March returned negative. His steroids were modified to oral prednisolone and tapered, and he was on oral antibiotic Cefdinir and oral anticoagulant Xarelto. His COVID-19 PCR on 4^th^ April was again negative. On day 41 of admission (19 days of EPO therapy), NIV was discontinued, and he tolerated NRBM alone without significant desaturation. His SpO_2_ maintained between 88-92% and his EPO therapy was gradually reduced to once per week until he was finally discharge on 5^th^ May. During follow-up, one week after home discharge, the patient had maintained oxygen saturation level above 94% with low flow oxygen (2-3 liter) by home concentrator.

**Patient perspective:** the patient was happy with the management and successful outcome of his infection.

**Informed consent:** a written informed consent was obtained from the patient for participation in this study.

## Discussion

The clinical presentation of patients with SARS-CoV-2 infection can range from asymptomatic disease, to mild respiratory tract infection, to severe pneumonia, to acute respiratory distress syndrome. The severity of the disease and mortality correlate with age, gender and prior comorbidities [5]. With very few effective antiviral therapy options available for treating patients with SARS-CoV-2 infection [6], management of severe cases is limited to supportive care measures, such as maintaining blood oxygen saturation and fluid management. Here, we report our experience with a case of severe COVID-19 in a previously healthy 43-year-old man. The patient presented with pneumonia, 70-80% lung involvement, severe hypoxia (PO_2_/FiO_2_ratio 49%) and type I respiratory failure. After failure to respond to standard interventions and continuing to deteriorate, the patient was administered EPO in an attempt to improve his O_2_ saturation. The patient responded positively within 24 hours and his treatment with EPO was continued. The patient made a full recovery and he was discharged on day 77 after admission.

Erythropoietin is a hormone, mainly secreted by the kidneys in response to cellular hypoxia and successfully used for the treatment of anemia. However, recent studies indicate that EPO has beneficial effects for a range of medical conditions, including patients in ICU [7]. Moreover, recent case studies suggested that EPO could also be used to ameliorate the clinical course and outcome of critically ill COVID-19 patients [2,8]. Although the exact mechanism of EPO action in COVID-19 is unclear, several possibilities have been suggested. Erythropoietin could exert its pleiotropic actions in the lung by protecting the integrity of the pulmonary epithelial and endothelial cells as well as by attenuating the associated pulmonary interstitial and alveolar epithelial edema and the deterioration of pulmonary oxygenation function. This could be via modulating multiple levels of early signaling pathways involved in apoptosis, inflammation and peroxidation, and potentially restoring overall homeostasis [4]. Another possibility is that EPO could counteract the inflammation and 'cytokine storm' observed in COVID-19 patients [9]. Pneumonia, lymphopenia, lymphocyte exhaustion markers and cytokine storm characterize severe COVID19. C-reactive protein and D-dimer are abnormally high. Substantially elevated serum levels of pro-inflammatory cytokines, including IL-6, IL-1β, IL-2, IL-8, IL-17, G-CSF, GM-CSF and others, contribute to shock and multi-organ damage [9,10]. Thus, suppressing the pro-inflammatory cytokines and enhancing the innate immune response to the virus, via triggering the induction of interferon, could help reduce the severity of the infection and improve respiratory function [3,4].

## Conclusion

Further studies are urgently required to assess EPO as a putative adjuvant in the treatment of severe COVID-19 cases.
